# Implementing a preimplantation proteomic approach to advance assisted reproduction technologies in the framework of predictive, preventive, and personalized medicine

**DOI:** 10.1007/s13167-022-00282-5

**Published:** 2022-05-21

**Authors:** Vasiliki Kanaka, Stavros Proikakis, Petros Drakakis, Dimitrios Loutradis, George Th. Tsangaris

**Affiliations:** 1 First Department of Obstetrics and Gynecology, School of Medicine, National and Kapodistrian University of Athens, Alexandra Hospital, Athens, Greece; 2 Proteomics Research Unit, Biomedical Research Foundation, Academy of Athens, Athens, Greece; 3 Third Department of Obstetrics and Gynecology, School of Medicine, National and Kapodistrian University of Athens, Attikon Hospital, Athens, Greece

**Keywords:** Predictive preventive personalized medicine (PPPM / 3PM), Assisted reproduction technology efficacy, Biomarkers, Individualized patient profile, Improved outcomes, Proteomics, In vitro fertilization, Assisted reproduction technology, Sperm, Oocyte, Embryo, Endometrium

## Abstract

The evolution of the field of assisted reproduction technology (ART) in the last 40 years has significantly contributed to the management of global infertility. Despite the great numbers of live births that have been achieved through ART, there is still potential for increasing the success rates. As a result, there is a need to create optimum conditions in order to increase ART efficacy. The selection of the best sperm, oocyte, and embryo, as well as the achievement of optimal endometrial receptivity, through the contribution of new diagnostic and treatment methods, based on a personalized proteomic approach, may assist in the attainment of this goal. Proteomics represent a powerful new technological development, which seeks for protein biomarkers in human tissues. These biomarkers may aid to predict the outcome, prevent failure, and monitor in a personalized manner in vitro fertilization (IVF) cycles. In this review, we will present data from studies that have been conducted in the search for such biomarkers in order to identify proteins related to good sperm, oocyte, and embryo quality, as well as optimal endometrial receptivity, which may later lead to greater results and the desirable ART outcome.

## Introduction

Infertility is an evolving global public health issue, affecting currently more than 186 million people, most of whom are residents of developed countries [1]. In 1990, infertility affected 42.0 million people, while in 2010, the number of people suffering from infertility had already risen up to 48.5 million [2]. Worldwide, there is an unequal distribution of the phenomenon, as 1 to 7 couples (15%), of reproductive ages, in the western world and 1 to 4 (25%) in the developing counties are affected. Interestingly enough, in some countries such as South Asia, countries in sub-Saharan Africa, the Middle East and North Africa, Central and Eastern Europe, and Central Asia, infertility rates can even reach up to 30% [1].

Infertility is defined as the inability to establish a clinical pregnancy after one year of regular unprotected intercourse and can either be primary, where no conception has previously occurred, and secondary, where, despite the occurrence of a previous clinical pregnancy, there is now an inability to establish one [3].

The causes of infertility can be classified into three major groups, including the male factor (30%), the female factor (30%), and combined and/or unexplained infertility (40%) [4]. The three major causes of female infertility are ovulation disorders (20%), tubal obstruction (20%), and endometriosis (5–10%). Other causes which can lead to infertility are pelvic inflammatory disease (PID) or uterine problems [5, 6]. Other risk factors which are related to female infertility include age, nutrition, weight, exercise, psychological stress, environmental and occupational exposures, cigarette smoking, illicit drug use, alcohol, and caffeine [7]. Male infertility is also classified into three major categories. These are defective spermatogenesis, defective transport, and ineffective delivery [8]. There are seven main causes of semen-related abnormalities that lead to the above dysfunctions. These include hormonal defects, physical reasons which can lead to disruption in sperm production and blockage of the ejaculatory pathway (varicocele 40%), sexual problems, environmental and lifestyle reasons, genetic factors, and epigenetic factors [9]. Finally, infertility can be caused by a combination of female and male factors, or no identifiable cause can be established.

Assisted reproduction technologies (ART) in the past 40 years, since the birth of the first in vitro fertilization (IVF) baby, Louise Brown in 1978 by Patrick Steptoe and Robert Edwards, have significantly contributed to the successful treatment of infertility worldwide [10]. Significant milestones, such as the utilization of donor oocytes in 1983, the introduction of Preimplantation Genetic Diagnosis (PGD) for sex-linked diseases in 1990, the first birth from IVF after Intracytoplasmic Sperm Injection (ICSI) in 1991, and the first birth from cryopreserved oocytes in 1997, have been major contributors to this journey [11]. In total, over 8 million IVF children have been born since the introduction of ART and over 2.5 million cycles are being performed annually, resulting in over 500,000 deliveries per year [12]. Alone, PGD has enormously contributed to the advancement of IVF success rates, as chromosome abnormalities which are detected through this technology are responsible for about 50% of early pregnancy losses [13, 14]. Despite these great numbers, IVF success rates remain suboptimal, having a 70% failure rate [10]. Moreover, a variation in the success rates of IVF clinics worldwide is also noticed. Based on annual data which are published by the European Society of Human Reproduction and Embryology (ESHRE) in Europe and the Centers for Disease Control and Prevention (CDC) in the USA, some top clinics have a success rate higher than 40%, while others have a success rate less than 10% [15]. The success rate is generally greater in women under 35, but usually, women treated with IVF are over 35 years of age [16].

The main method currently used to assess the quality of the embryos and to increase the chance of a successful IVF procedure is the morphological evaluation of the embryo. Morphological evaluation of the embryo is done under light microscopy [17]. Despite its wide application, the method has limitations which are firstly the subjective evaluation and secondly the exposure to pH, light, and temperature shifts, which all have negative effects on the embryo’s development and quality [18]. Time-lapse technology (TLT), on the other hand, which has tried to replace conventional morphological evaluation, is a method that has managed to increase the number of observations and provides a dynamic assessment of developing embryos. TLT offers an uninterrupted culture environment; it minimizes embryo handling as well as the need to expose embryos to conditions outside the incubator [19]. The increased expense of equipment, however, consumable materials and extra space are considered its drawbacks. Despite TLT’s advantages, there is currently insufficient evidence that it is superior to conventional morphological assessment [20]. Taking as a result into consideration the fact that there are numerous factors contributing to good embryo quality besides morphology, there is a need for further investigation of the gametes, embryo, and uterine environment in order to achieve optimal clinical ART outcomes.

The field of proteomics, which is a recently developed field, could assist in the optimization of ART clinical outcomes, as it can provide the ART community with important insights at the molecular level of physiological processes during reproduction by studying all the interactions between the sperm, the oocyte, the embryo, and the uterus. Proteomics utilizes the application of technologies for the identification and quantification of overall proteins that participate in the above interactions and processes [21]. In such a way, they identify the alterations in the expression profile of key proteins (biomarkers), which could then be utilized for predicting the possible outcome of an IVF cycle, preventing an unsuccessful attempt of IVF, and monitoring the cycle in a personalized manner in order to achieve the desired outcome [22]. This approach is also the main scope of predictive, preventive, and personalized medicine [23]. A lot of proteomic studies have been conducted in an effort to investigate and better comprehend the molecular pathways behind human reproduction. The studies have been focused on protein profiles of human gametes, human embryo, the endometrium, and their correlation to the IVF outcome.

## Semen proteomics

Proteomic analysis of semen can be conducted using either the spermatozoa itself or the seminal plasma. Semen actually consists of 5% spermatozoa (SPZ) and 95% of seminal plasma (SP) [24]. Seminal plasma represents the non-cellular liquid constituent of semen, which mainly contains sugars, oligosaccharides, glycans, lipids, inorganic ions (calcium, magnesium, potassium, sodium, and zinc), and small metabolites [25]. SP originates from the seminal vesicles (65%), the prostate (25%), the testis, and the epididymis (10%). Aside from soluble proteins, the seminal plasma also includes proteins in microvesicles, released from the epididymis (epididymosomes) and prostate (prostasomes) [26]. Normally, as sperm moves through the male and female reproductive tract, it goes through the process of maturation. More precisely, after sperm is released from the seminiferous tubules in the testis, it passes through the epididymis, where it acquires its forward progressive motility. However, it is until it reaches the female reproductive tract that it completes its maturation through the processes of capacitation and acrosome reaction. Sperm capacitation is referred to the acquisition of hyperactive motility, while the acrosome reaction is the release of acrosomal contents from the spermatozoa, a process needed for the penetration of zona pellucida and fusion with the oocyte membrane [27]. A landmark of capacitation is considered to be protein tyrosine phosphorylation, which occurs during the late stages of capacitation. Most of the Tyr-phosphorylated proteins include ion channels, metabolic enzymes, and structural proteins [27].

### Proteomic profile of normal sperm

Many studies have analyzed the proteomic profile of normal semen. Yuan li *et al.* performed two-dimensional polyacrylamide gel electrophoresis (2DE-PAGE) matrix-assisted laser desorption ionization time of flight MS analysis in healthy testes and found a total of 725 unique proteins, 525 of which were present in the sperm-milieu and 319 on the spermatozoa. Out of the 319 proteins, 47% were intrinsic sperm proteins, while 23% were extrinsic, originated from the epididymis, probably also acquired during the maturation process. Among them were proteins involved in metabolism, immune defense, structural proteins, antioxidants, proteins involved in reproduction, signal transduction, and others [28]. Guo *et al*. did an attempt to study the proteomic composition of spermatozoa. He applied 1-D SDS-PAGE (one-dimensional sodium dodecyl sulfate PAGE) and reversed-phase liquid chromatography–mass spectrometry (RP-LC-MS/MS) in human testis samples. A plethora of proteins was detected originating from the nucleus, cytoplasm, and membrane of the human testis. More precisely, he identified 39 testis-specific proteins. A percentage of 7% of these proteins originated from the mitochondrial, 4% from the endoplasmic reticulum, 3% from the Golgi apparatus, and 1% were proteins of the lysosomes. With regards to their biological roles, there was a significant number of proteins related to spermatogenesis and androgen production such as cell cycle, apoptosis, steroid biosynthesis, and regulation of translation. Baker *et al.* analyzed the head and flagella proteome of human sperm with SDS-PAGE and LC-MS/MS and found 1429 proteins. Among them, 179 were common in the head and tail. These proteins were mainly related to energy production. More specifically, proteins of the oxidative phosphorylation group, glycolysis, citric acid cycle, fatty acid synthesis, β-oxidation group, amino acid metabolism, gluconeogenesis, steroid metabolism, and amino acid catabolism were identified in the flagella, and proteins of glycolysis, fatty acid synthesis, β-oxidation, amino acid metabolism, steroid metabolism, and amino acid catabolism were identified in the head [29]. The presence of proteins related to metabolism and energy production in the sperm tail was also shown by Amaral *et al.*, who analyzed the proteome profile of the human sperm tail after performing liquid chromatography and tandem mass spectrometry. A total of 1049 proteins were identified. Among them were also some proteins related to sperm tail structure [30, 31]. Finally, in the study of Mateo *et al.*, who attempted to analyze sperm nuclear proteins by performing 2-DE and 1-DE, followed by LC-MS/MS in sperm samples (ejaculates), 403 proteins were identified. The most abundant family among these proteins was that of histones, followed by the ribosome proteins, proteasome subunits, cytokeratins, tubulins, SPANX proteins (sperm protein associated with the nucleus on the X chromosome), HSPs (heat shock proteins), and tektins [32].

### Proteins involved in sperm maturation

Several studies have been conducted with the aim to better comprehend the protein pathways involved in normal sperm capacitation and acrosome reaction. As mentioned above, a key step in sperm capacitation is the phosphorylation of sperm proteins. Ficarro *et al.* analyzed with 2DE gel analysis coupled with antiphosphotyrosine immunoblots and tandem mass spectrometry (MS/MS), the phosphoproteome of human capacitated sperm, in order to study the proteins that normally participate in that process, and their localization in the SPZ. Among their results, valosin-containing protein (VCP), a homolog of the SNARE-interacting protein NSF which is a member of the AAA family (ATPases associated with various cellular activities), which normally mediates the fusion of Golgi membranes and two members of the A kinase-anchoring protein (AKAP) family (AKAP-3, AKAP-4), which are involved in signal transduction in discrete regions of the cell, were found to be tyrosine phosphorylated during capacitation. Immunolocalization of VCP showed fluorescent staining in the neck of non-capacitated sperm, while after capacitation, the staining on the neck was decreased and increased on the anterior head [33]. Wang *et al*., in the same manner, tried to analyze the overall phosphorylation events during sperm capacitation in human sperm after label-free quantitative phosphoproteomics, and they managed to identify 231 sites with increased phosphorylation levels. Their results showed that the activity of tyrosine phosphorylation kinases, specifically on insulin growth factor 1 receptor (IGF1R), is remarkably upregulated during capacitation which can indicate that the IGF1R-mediated tyrosine phosphorylation pathway may serve as a key player in the regulation of sperm capacitation and could be a target for improvement of sperm functions in infertile men [34]. Moreover, the study of Hernandez-Silva *et al*. attempted to analyze the human sperm plasma membrane-associated proteins (SMAP) and their role during sperm capacitation. They used 2DE and MS and found twenty-nine proteins, all of which have already been identified in the human seminal fluid. The study showed that the association of SMAP proteins with sperm plasma membrane affects protein phosphorylation and, as a result, sperm hyperactivation, which is part of the capacitation process [35]. Castillo et al. analyzed the proteomic changes that sperm undergoes in preparation for fertilization using isotopic peptide labeling and liquid chromatography, followed by tandem mass spectrometry. Thirty-six proteins were found to undergo significant changes in their relative abundance, among them, Erlin-2 (ERLIN2), gamma-glutamyl hydrolase (GGH), and transmembrane emp24 domain-containing protein 10. The above proteins were categorized in the following functional groups which were sperm motility, fertilization, energy production, signaling, detoxification/antioxidant response, protein degradation, protein folding, vesicular trafficking, metabolism of folic acid, and RNA biogenesis [36].

### Potential biomarkers for male infertility

Male infertility can be a result of several causes. However, a percentage of about 70% of male infertility cases are of unknown origin [37]. Irrespective of the cause, clinical forms of male infertility can be oligospermia (reduced sperm counts), asthenozoospermia (reduced sperm motility), teratospermia (abnormal sperm morphology), and azoospemia (complete lack of spermatozoa in semen). Azoospermia affects 5–20% of infertile men and can be classified as non-obstructive azoospermia (NOA) or pre-testicular and obstructive azooospermia (OA). NOA is caused by hypothalamic or pituitary dysfunction, which results in low serum levels of follicle-stimulating hormone (FSH) and luteinizing hormone (LH), which further lead to low testosterone levels and failure of the testes to produce sperm; NOA can be further categorized to maturation arrest (MA), Sertoli cell-only syndrome (SCO), and hypospermatogenesis (HS). OA, on the other hand, is a result of a physical obstruction in the male genital tract. Finally, an additional cause of male infertility affecting 5–6% of infertile men is the production of sperm antibodies in semen or blood which usually results in the reduction of sperm motility and prevention of sperm-egg binding during fertilization [38]. The proteomic studies that have analyzed the differences in the expression of protein profiles between fertile and non-fertile patients could lead to the identification of biomarkers that may help in the early detection of infertile men as well as in their treatment in a more personalized manner [39].

Numerous studies have been conducted searching for biomarkers indicating male infertility. Starting with the studies that compared fertile and infertile semen, without specifying the underlying cause, Zhu *et al.* compared semen samples that had a positive IVF/AID (artificial insemination by donor) outcome with those that did not, through HPLC-MS/MS, and found twenty-one proteins to be differentially expressed between the two groups. Five proteins were overexpressed in the group that did not result in pregnancy, which were A2LD1 (gamma glutamylamine cyclotransferase), CRISP2 (cysteine-rich secretory protein 2 precursor), ATP1B3 (sodium/potassium-transporting ATPase subunit beta-3), PGRMC1 (progesterone receptor membrane component 1), and Fbxo2 (F-box only protein 2). A2LD1 plays a role in the degradation of cross-linked proteins by transglutaminases, while CRISP family proteins and PGRMC1 are secreted at different sites in the epididymis and seminal vesicle; ATP1B3 is a Na/K-ATPase beta-3 polypeptide that maintains an electrochemical gradient across the plasma membrane, while FBX02 constitutes one of the four subunits of the ubiquitin-protein ligase complex called SCFs. The ubiquitin-proteasome system targets proteins for degradation [40]. Thacker et al. also compared the proteome of fertile and infertile semen with LC-MS/MS and found four unique proteins to be present only in the semen of fertile men. These were SEMG2 pre (semenogelin 2 precursor), PIP (prolactin-induced protein), CLU isoform1 (clusterin isoform 1), and PSA isoform 1 pre (prostate-specific antigen isoform 1 preproprotein). The above proteins have an important role in sperm capacitation [41]. Xu *et al*. performed 2DE (2-dimensional electrophoresis) coupled with MALDI-TOF/TOF to analyze infertile semen and identified 24 differentially expressed proteins. Upregulated proteins were ANXA5 (annexin A5), PSA (prostate-specific antigen), PSA pre (prostate-specific antigen precursor), CLU (clusterin), SEMG1 (semenogelin 1), SEMG1 pre (semenogelin 1 precursor), SMG2 (semenogelin 2), and SEM2pre (semenogellin2 precursor), and downregulated were AKAP4 (A kinase anchor protein 4), AKAP4 pre (A kinase protein 4 precursor), ODFP (outer dense fiber protein), SPANX (sperm protein associated with nucleus on the X chromosome), proteasome subunit alpha type 1, (PSMA1), and PIPpre (prolactin-inducible protein precursor). The proteins mainly belonged to sexual reproduction, response to wounding, metabolic processes, cell growth, and/or maintenance groups [42]. In the study of Martins et al., seminal plasma of fertile and infertile men with primary and secondary infertility was compared. Proteomic shotgun and bioinformatic analysis were applied, and it was revealed dysregulation of the biological processes of cell secretion in primary infertility and vesicle-mediated transport as well as a dysregulation of the immune system response, of proteolysis and iron homeostasis in secondary infertility. After western blotting was performed, ANXA2 (annexin A2) and CDC42 (cell division control protein 42 homolog) were found to be overexpressed, while SEMG2 (semenogelin-2) underexpressed in primary infertility, and proteins ANXA2 (annexin A2) and APP (amyloid precursor protein) were overexpressed in secondary infertility and could potentially serve as biomarkers in the identification of infertility [43]. Pixton et al. compared the proteome profile of sperm from one patient who experienced failed fertilization in IVF with three fertile donors. After performing 2DE and MS/MS, he found 20 proteins to be differentially expressed among the two groups. The most significant differences were in the secretory actin-binding protein (SABP) and dense fiber protein 2/2 (ODFP), which were overexpressed in the infertile sperm. SABP interacts with CD4 molecules on T-cells that are involved in immune response, while ODFP has a structural role [44]. Finally, in the study of Selvam *et al.*, pooled semen samples of eight fertile and nine infertile men were compared after the performance of LC-MS/MS and 162 proteins were differentially expressed among the two groups. After the performance of western blotting, it was found that protein ANXA2 (annexin A2) was overexpressed and SPA17 (surface protein Sp17) and SPI (serine protease inhibitor) were underexpressed in men with unexplained infertility. ANXA2 is a structural protein, Sp17 is a cell surface protein, and SPI is an enzyme which antagonizes serine protease activity [45].

### Proteomics in oligoasthenospermia/asthenospermia

A lot of research has been conducted on poor semen motility. The significance of the same protein (SABP) and its relation to poor sperm quality was also indicated in the study of Capkova *et al*., where sperm samples of fertile and non-fertile men were studied by western blotting and matrix-assisted laser desorption/ionization mass spectrometry and SABP protein was found to be overexpressed in asthenospermia or oligoasthenospermia compared to normal semen. The highest localization cite of the protein was the tail, specifically the midpiece [46]. A study by Siva et al. that compared normal sperm motility with asthenospermia using two-dimensional PAGE MALDI MS/MS analysis found eight proteins to be differentially expressed between the two groups. Specifically, it was found that PSMA3 (proteasome subunit alpha type 3), HSPA2 (heat shock 70 kDa related protein), TUBB2C (tubulin beta-2C chain), TEKT1 (tektin I), and one of Mr ~33kDa and pL ~5.7 protein were lower in patients with asthenospermia, while TPIS (triose-phosphate isomerase), GKP2 (testis-specific glycerol kinase 2), and OXCTI (succinyl-CoA:30ketoacid co-enzyme A transferase I, mitochondrial precursor) were higher in patients with asthenospermia than in patients with normospermia. These proteins were distributed to three functional groups: energy and metabolism (TPIS, GKP2, and OXCTI), movement and organization (TUBB2C and TEKT1), and protein turnover, folding, and stress response (PSMA3 and HSPA2) [47]. Parte *et al*. also compared the two groups (asthenospermia with normospermia) with Nano LC-MS and identified 66 phosphoproteins differentially regulated in asthenospermia (39 upregulated: prostate-specific antigen (PSA), ubiquitin, and 27 downregulated: SPANXB1, ODF1, PIP, AKAP4). These deregulated proteins had various roles, including HSPs, cytoskeletal proteins, proteins associated with the fibrous sheath, and those associated with energy metabolism. Four proteins were unique in patients with asthenospermia. These were phospholipase A2 membrane-associated (PLA2G2A), myosin regulatory light chain 12A (MYL112A), rho GDP dissociation inhibitor 1 (ARHGHIA), and tubulin alpha 1Bchain (TUBA1B) [48]. In the study of Martinez-Heredia *et al.*, where two-dimensional electrophoresis was performed, seven proteins were identified in a lower amount in the patients with asthenospermia compared to normal semen. These were actin b (ACTB), annexin a5 (ANXA5), cytochrome-C oxidase subunit-6B (COX6B), histoneH2A, prolactin-inducible protein (PIP), prolactin-inducible protein precursor (PIPpre), and calcium-binding protein-S100A9. At the same time, other proteins were detected in higher amounts. These were clusterin precursor (CLUpre), dihydrolipoamide dehydrogenase precursor (DLDpre), fumarate hydratase precursor (FHpre), heat shock protein-HSPA2 (HSPA2), inositol-1 monophosphatase (IMPA1), proteasomesubunit-PSMB3 (PSMB3), semenogelin 1 precursor (SEMG1pre) and testis-expressed sequence 12 (TEX12), 3 mercvhaptopyruvate sulfurtransferase, and dienoyl-CoA isomerase precursor. The above proteins are involved in the functional groups of energy production, cell signaling and regulation, structure, and movement [49]. The group of Hashemitabar *et al****.*** performed 2DE and MALDI-TOF-TOF in order to compare normospermia with asthenospermia in semen samples. He found 14 unique proteins, which were tubulin beta 2B (TUBB2B), glutathione S-transferase Mu 3 (GST Mu3), keratin, type II cytoskeletal 1 (krt1), outer dense fiber protein 2 (ODF2), voltage-dependent anion-selective channel protein 2 (VDAC2), A-kinase ancho protein 4 (AKAP4), cytochrome c oxidase subunit 6B (COX6B), sperm protein associated with the nucleus on the X chromosome B (SPANXB), phospholipid hydroperoxide glutathione peroxidase-mitochondrial (PHGPx), isoaspartyl peptidase/L-asparaginase (ASRGL1), heat shock-related 70kDa protein 2 (HSPA2), stress-70 protein mitochondrial (HSPA9), glyceraldehyde-3-phosphote dehydrogenase testis-specific (GAPDS), and clusterin (CLU). The above proteins belong to groups of structural proteins, proteins involved in zona reaction, in glycolysis, sperm motility, oxidative stress, ATP synthesis, and inflammation [50]. Wang *et al*. analyzed the seminal plasma of men with asthenospermia using gel electrophoresis and in-gel digestion coupled with liquid chromatography-mass spectrometry (LC-MS/MS) and found 45 upregulated and 56 downregulated proteins, most of which were proteins of the epididymis and the prostate. This finding can indicate that abnormalities of the epididymis and prostate can also lead to infertility. It was identified that DJ-1 protein (protein Deglycase) was downregulated in seminal plasma of patients with asthenospermia, which is shown to be involved in the regulation of oxidative stress [51]. Giacomini *et al.* analyzed in their study seminal plasma samples of patients with oligoasthenospermia and normospermia with two-dimensional gel electrophoresis and nano-liquid chromatography-electrospray ionization-mass spectrometry/mass spectrometry. He identified four proteins differentially expressed. Two of them [epididymal secretory protein E1 (NPC2, HE1) and galectin-3-binding protein (Gal-BP)] were underexpressed, and the other two [lipocalin-1 (LCN1) and a prolactin-inducible protein form (PIP)] were overexpressed in the group with oligoasthenospermia. NPC2 is a major component of epididymal secretions and functions as a cholesterol transporter and a regulator of cholesterol homeostasis, while M2BP is involved in cell-cell and cell-matrix interactions. Lipocalin 1 is a glycosylated secretory protein present in fluids covering epithelial surfaces. It scavenges lipids, negatively regulates cysteine proteinases, and exerts antimicrobial activity by trapping bacterial siderophores. PIP is a glycosylated, secreted glycoprotein present in a number of body secretory fluids and prostasomes; it is reported to bind to the post-acrosomal rein of spermatozoa [52]. Other attempts were also made to identify proteins that are related to sperm motility. Zhao *et al.*, after performing two-dimensional electrophoresis, followed by MALDI-TOF, identified ten proteins which fall into three categories: structure-associated proteins, metabolic enzymes, and three other functional proteins. These were rho GDP dissociation inhibitor (Rho DGI), outer dense fiber protein (ODFP), isocitrate dehydrogenase subunit a (NADsubg), phosphoglycerate mutase 2 (PGAM2), triose-phosphate isomerase (TPI), glutamate oxaloacetate transaminase-1 (GOT1), carbonic anhydrase II (CAII), semenogelin-1 precursor (SEM1pre), glutamine synthase (GS), and 26S protease regulatory subunit (PSMC2). Half of the proteins identified were enzymes associated with sperm energy metabolism as the spermatozoon needs to consume ATP which is mainly produced through glycolysis and oxidative phosphorylation. Isocitrate dehydrogenase subunit a (IDH-α) was also identified, which is a key enzyme in tricarboxylic acid cycle (TAC). Low expression of IDH may disrupt sperm motility [53]. Martin-Hidalgo *et al*. compared low motility with high motility spermatozoa with nano HPLC-MS/MS triple TOF and found that the majority of phosphoproteins that are present in low motility spermatozoa were involved in sperm metabolism, while proteins in spermatozoa with high motility were associated to spermatogenesis and metabolism. One of the most abundant phosphoprotein that is present in sperm with high motility is glycogen synthase kinase 3a (GSK3a) which is actually a kinase that regulates motility in sperm [54]. In the study of Shen *et al*., the analysis of sperm samples with asthenospermia was done using two-dimensional electrophoresis followed by mass spectrometry and a total of sixteen proteins were identified which belonged to 15 unique protein groups. These were GRP78 (glucose-regulated protein 78), Lactoferrin, SPANXB (sperm protein associated with the nucleus, X-linked, family member B), PGK2 (phosphoglycerate kinase 2), flagellin, DJ-1 protein, XPA binding protein 2, CAB2 (XPA binding protein 2, isoform CRA_b), GPX4 (phospholipid hydroperoxide glutathione peroxidase), and GAPDH (glyceraldehyde-3-phosphate dehydrogenase, testis-specific). The above proteins are involved in the HSP family, are constituents of the cytoskeleton, play a role in immune response, spermatid development, sperm motility, single fertilization, binding, and oxidative stress [55].

### Proteomics in azoospermia

In the study of Batruch *et al*., analysis of seminal plasma from patients with non-obstructive azoospermia (NOA) was performed with liquid chromatography coupled with mass spectrometry and a total of 2048 proteins were found; the majority of which are cytoplasmic, membrane, and extracellular. Most of them are involved in catalytic activity and many others in protein binding. Some of them are enzymes. With regards to the differentially expressed proteins, 98 proteins had different abundance in the two groups; among these findings, isoform 1 of gamma-glutamyltransferase 7 (GGT7) was unique in the NOA group, alpha-2-macroglobulin (A2M), semenogelin-2 (SEM2G) were lower in NOA, isoform 3 of guanine nucleotide exchange factor VAV2 (RhoGEF VAV2) and isoform 1 of protein-glutamine gamma-glutamyltransferase 2 (TGM2) were higher in NOA [56]. Li *et al*. analyzed the testis samples of patients with azoospermia due to Sertoli cell-only syndrome with two-dimensional gel electrophoresis, followed by MALDI-TOF/TOF MS, and found thirteen differentially expressed proteins in comparison with the normal group. Among them was protein heterogenous nuclear ribonucleoprotein L (HnRNPL) which was downregulated in the abnormal sperm and is normally involved in functions such as apoptosis and growth in spermatogenic cells [57]. In the study of Drabovich *et al*., who performed a selected reaction monitoring assay between sperm samples with azoospermia and normal ones, two differentially expressed proteins were identified. These were epididymis-expressed ECM1 (extracellular matrix protein 1), which was overexpressed in samples with NOA and underexpressed in samples with OA. On the other hand, testis-expressed TEX101 (testis-expressed protein 101) was underexpressed in OA and NOA groups compared to the control group. The first is an extracellular matrix protein with structural function, while the exact function of TEX101 is yet unknown [58]. Yamakawa *et al.* compared seminal plasma from fertile and non-fertile men with 2-dimensional electrophoresis and LC-MS/MS and found 4 proteins (stabilin 2 STAB2, 135-kD centrosomal protein, CP135, guanine nucleotide-releasing protein GNRP, and prolactin-inducible protein PIP) as potential biomarkers for NO azoospermia and 1 protein [epididymal secretory protein E1 (NPC2)] as a potential biomarker for obstructive azoospermia. STAB, CP135, and GNRP exist as a membrane or intracellular proteins, while PIP is probably secreted from the epididymis and the testis. NCP2 is a major secretory protein of the epididymis [59]. In the study of Heshmat et al., after measurement of the concentration of L-PGD synthase (lipocalin type prostaglandin D synthase) in seminal plasma with highly sensitive and specific noncompetitive immunoassay, it was found that the protein was downregulated in the group with obstruction. Thus, LPGDS could be a potential biomarker for assessing patency in the seminal tract, although its function is still unclear [60]. In the study of Davalieva *et al***.,** seminal plasma of four different groups was analyzed with two-dimensional gel electrophoresis coupled with MS. The groups were normospermia, asthenospermia, oligospermia, and azoospermia groups. Eight proteins were found to be upregulated in the azoospermia group in comparison to the other groups. These proteins were fibronectin (FN), prostatic acid phosphate (PAP), proteasome subunit alpha type-3 (PSMA3), beta-2-microglobulin (BM2), galectin-3-binding protein (GAL-3BP), prolactin-inducible protein (PIP), and cytosolic nonspecific dipeptidase (CNDP). No statistically significant difference in protein expression was found between the groups of normospermia, oligospermia, and asthenospermia. They suggest PAP as a potential strong biomarker for azoospermia, which is also overexpressed in prostate carcinoma, as it is normally secreted from the gland [61].

### Proteomics in teratospermia

Netherton *et al*. analyzed nuclear extracts of human semen comparing high- and low-quality semen using LC-MS/MS and found that nuclear retention of specific proteins is a common facet among low-quality sperm cells. In particular, it was found that the presence of the enzyme topoisomerase 2A (TOP2A) in the sperm head is highly correlated to poor head morphology, and as a result, it could serve as a potential biomarker for confirming male infertility in clinical practice [62]. Vigodner *et al*. researched the occurrence of the post-translational modification, sumoylation, in normal and abnormal sperm samples. Sumoylation is the addition of small ubiquitin-like modifiers (SUMO) to other sperm proteins. After the performance of immunofluorescence and electron microscopy, it was found that small ubiquitin-like modifiers (SUMO) SUMO1 and SUMO2/3 were localized in the neck area of human sperm and were also detectable in the flagella and some head regions. These SUMO proteins were in higher concentrations in the neck and tail region of nonmotile, two-tailed, curled tailed, misshapen, microcephalic (small head), and acephalic (no head) sperm in comparison to normal sperm. After the performance of western blotting and mass spectrometry, 55 SUMO targets corresponded to flagella proteins, proteins involved in the maturation and differentiation of sperm, heat shock proteins, and glycolytic and mitochondrial enzymes. Among these proteins were heat shock-related 70 kDa protein 2, outer dense fiber protein 3 (ODFP3), A-kinase anchor proteins 3 and 4 (AKAP3, AKAP4), L-lactate dehydrogenase C, sperm protein associated with the nucleus on the X chromosome B/F (SPANXB/F), valosin-containing protein, semenogelins, histone H4, and ubiquitin [63]. The proteomics of abnormal sperm morphology has also been studied by Wang *et al*. who performed two-dimensional electrophoresis, followed by mass spectrometry, comparing fertile and infertile semen samples from artificial insemination cycles done by donor sperm and identified 26 proteins which were differentially expressed and were mainly related to biological processes like sperm motility, energy consumption, and structure. Among these proteins were sperm proteins associated with the nucleus on the X chromosome (SPANX) proteins, whose function is unknown, but they were found to be downregulated in the low fertility group. Interestingly, it is speculated that ROS produced by stress or abnormal condition affects SUMOlyzation of SPANX protein, which leads to the increase of sperm DNA damage and as a result impairs fertility [64].

Sharma *et al.* studied the differences in proteins between four groups of sperm samples: normal sperm count and morphology, normal sperm count with abnormal morphology, oligozoospermia with normal morphology, and finally oligozoospermia with abnormal morphology using liquid chromatography and mass spectrometry. They found 24 proteins differentially expressed between the four groups; 3 were downregulated in the normal sperm count with abnormal morphology group [mucin 6 gastric (MUC6 gastric), orosomucoid 1 precursor (ORM1pre), and acidic epididymal glycoprotein-like isoform 1 precursor 1 (AEG like protein1)], 1 in the oligozoospermia with abnormal morphology group [clusterin 1, (CLU1)], 2 were upregulated in the oligozoospermia with normal morphology (zinc alpha-2 glycoprotein 1 (AZGP1) and tissue inhibitor of metalloproteinase 1 precursor (TIMP1), and 2 were upregulated in the oligozoospermia with abnormal morphology group (prostate-specific antigen isoform 1 preprotein (KLK3), semenogelin 1 isoform b preprotein (SEMG1b pre). Their functions include response to stress, transport, developmental process, lipid metabolic process, protein maturation, and others [65]. Finally, Kanannejad and Gharesi-Fard studied unexplained male infertility in seminal plasma samples from men undergoing in vitro fertilization with 2D-PAGE, followed by mass spectrometry, and found two differentially expressed proteins clusterin (CLU) and epididymal secretory protein E1 (NPC2), which is a major component of epididymal secretion, being overexpressed, while prostate-specific antigen was downregulated in the group which succeeded in the IVF process [66].

Among the basic cellular pathways that play a pivotal role in normal sperm function and infertility are cytoskeleton organization/structural proteins and energy metabolism (especially in oligoasthenospermia/asthenospermia), protein degradation (apoptosis), regulation of oxidative stress, and immune response pathway. All the forementioned potential biomarkers of male infertility are presented in Table 1. Discovering biomarkers that indicate association with male infertility could help in either prevent earlier an unwanted unsuccessful result of and IVF cycle or even monitor each cycle by a prospective therapeutic invasive approach.

**Table 1:** Potential biomarkers of male infertility

Study group	Protein	Role	Up-/downregulated, unique in group	References
No pregnancy group	CRISP2 PGRMC1 A2LD1 ATP1B3 Fbxo2	Αpoptosis, SP constituents	Up	[40]
Fertile group	SEMG2pre, CLU1, PIP, PSA isoform 1 pre	Sperm capacitation	Unique	[41]
Infertile group	ANXA5 PSA PSApre CLU SEMG1, SEMG1 pre, SMGII SEMIIpre	Sexual reproduction, response to wounding, metabolic processes, cell growth and/or maintenance	Up	[42]
	AKAP4 AKAP4pre, ODFP, SPANX PSMA1, PIPpre	Structural, binding, sperm capacitation	Down	[42]
Primary infertility	SEMG2	SP coagulum constituent	Down	[43]
	ANXA2, CDC42	Membrane transport	Up	[43]
Secondary infertility	ANXA2, APP	Membrane transport, iron homeostasis	Up	[43]
Infertile group	SABP, ODFP	Immune response, structure	Up	[44]
Asthenospermia/oligoasthenospermia	SABP protein	Immune response	Up	[46]
Asthenospermia	PSMA3, HSPA2, TUBB2C, TEKT1	Energy and metabolism, folding and stress response	Down	[47]
	TPIS, GKP2, OXCTI	Movement and organization	Up	[47]
Asthenospermia	PLA2G2A, MYL112A, ARHGHIA, TUBA1B	HSPs, cytoskeletal proteins, energy metabolism	Unique	[48]
	SPANXB1, ODF1, PIP, AKAP4	Fibrous sheath, capacitation, binding	Down	[48]
	PSA, ubiquitin	Apoptosis	Up	[48]
Asthenospermia	ACTB, ANXA5, COX6B, histone H2A, PIP, PIPpre, calcium-binding protein-S100A9	Structure, cell signaling and regulation, energy production	Down	[49]
	CLUpre, DLDpre, FHpre, HSPA2, IMPA1, PSMB3, SEMG1pre, TEX12	Energy production, structure and movement, signaling and regulation	Up	[49]
Asthenospermia	TUBB2B, GST Mu3, KRT1, ODF2, VDAC2, AKAP4, COX6B, SPANXB, PHGPx, ASRGL1, HSPA2, HSPA9, GAPDS, CLU	Structural proteins, zona reaction, glycolysis, motility, oxidative stress, ATP synthesis, inflammation	Unique	[50]
Asthenospermia	DJ-1 protein	Oxidative stress	Down	[51]
Oligoasthenospermia	NPC2, Gal- BP	cholesterol transport/homeostasis, cell-cell and cell-matrix interactions	Down	[52]
	LCN1, PIP	Scavenge lipids, negatively regulates cysteine proteinases and exerts antimicrobial activity by trapping bacterial siderophores; binding to post-acrosomal region of spermatozoa	Up	[52]
Low sperm motility	Rho DGI, ODFP, NAD sub g, PGAM2, TPI, GOT1, CAII, SEM1pre, GS, PSMC2	Structure-associated proteins, metabolic enzymes	Up	[53]
Low sperm motility	IDH-a	Cellular homeostasis	Down	[53]
High motility	GSK3a	Kinase, involved in sperm motility	Up	[54]
Asthenospermia	GRP78, lactoferrin, SPANXB, PGK2, flagellin, DJ-1 protein, XPA binding protein 2, CAB2, GPX4, GAPDH	HSP family, constituent of cytoskeleton, immune response, spermatid development, sperm motility, single fertilization, binding, oxidative stress	Unique	[55]
NOA	GGT7	Catalytic activity followed, protein binding, enzymes	Unique	[56]
	A2M, SEM2G	Inhibition of fibrinolysis, sperm maturation	Down	[56]
	RhoGEF VAV2, TGM2	Angiogenesis, wound healing, cellular differentiation	Up	[56]
Azoospermia	hnRNP L	Apoptosis and death, growth in spermatogenic cells	Down	[57]
NOA	ECM1	Structure	Up	[58]
	TEX101	Unknown	Down	[58]
OA	ECM1, TEX101	Structural, unknown	Down	[58]
NOA	STAB2, CP135, GNRP, PIP	Membrane or intracellular proteins; epididymis/testis secretion	Unique	[59]
OA	NPC2	Epididymis secretion	Unique	[59]
OA	LPGDS	Unclear	Down	[60]
Azoospermia	FN, PAP, PSMA3, BM2, GAL-3BP, PIP, CNDP	Increased expression in prostate carcinoma (PAP)	Up	[61]
Poor morphology	TPO2A	Enzyme	Up	[62]
Infertile group	SPANX proteins	Unknown	Down	[64]
Unexplained infertility	ANXA2	Structural	Up	[45]
Unexplained infertility	SPA17, SPI	Surface protein; enzyme which antagonizes serine protease activity	Down	[45]
Oligospermia	MUC6 gastric, ORM1pre, AEG like protein1	Response to stress, transport, developmental process, lipid metabolic process, protein maturation and others	Down	[65]
Oligospermia, abnormal morphology	KLK3, SEMG1b pre	Sperm maturation	Up	[65]
Oligospermia	AZGP1, TIMP1	Lipolysis, extracellular matrix (ECM) composition	Up	[65]
Oligospermia, abnormal morphology	CLU1	Sperm maturation	Down	[65]
Unexplained infertility	CLU, NPC2	Sperm maturation, oxidative stress-induced apoptosis, agglutination of abnormal spermatozoa and complement-mediated sperm lysis; major component of epididymal secretion	Down	[66]
Unexplained infertility	PSA	Secretion of prostate epithelial cells	Up	[66]

## Oocyte proteomics

The aim of the proteomic analysis of the oocyte environment is an in-depth comprehension of the molecular pathways behind oocyte maturation, development, and competence acquisition. The identification of protein pathways that participate in the communication between oocytes, cumulus cells, and the follicular fluid may be significant for the identification of key proteins which could serve as biomarkers indicating good oocyte quality. Most proteomic studies that have been conducted have focused on the analysis of the follicular fluid and oocyte surrounding granulosa cells (cumulus cells).

### Proteomic analysis of follicular fluid

The follicular fluid (FF) is the natural environment in which the oocyte matures and becomes competent. Until now, only a small portion of the entire human FF proteome has been revealed. FF, due to its proximity and communication with the maturing oocyte, makes up a unique fluid for the study of the processes occurring during oocyte maturation [67]. Some studies were conducted to comprehend the molecular pathways that are involved in normal oocyte development through the proteomic analysis of the follicular fluid. In the study of Ambekar *et al.*, the proteome of human FF was analyzed by SDS-PAGE, OFFGEL, and strong cation exchange (SCX)-based separation, followed by LC-MS/MS and 480 proteins were identified. These proteins of the FF belonged to functional categories such as growth factors, hormones, receptor signaling, enzyme catalysis, defense/immunity, and complement activity [68]. In the study of Twigt *et al*., after proteomic analysis of FF with SDS-PAGE, in tube gel digestion and prefractionation of proteolytic peptides, followed by LC-MS/MS, 246 proteins were identified, most of which are involved in coagulation and immune response pathways [69]. Moreover, a study done by Jarkovska *et al.*, who performed two-dimensional gel electrophoresis, followed by MALDI mass spectrometry, showed that FF consists of proteins involved in the complement cascade, angiogenesis, and coagulation cascade [70]. Shen *et al.* performed reverse-phase high-performance liquid chromatography (RP-HPLC), followed by matrix-assisted laser desorption/ionization time of flight tandem mass spectrometry (LC-MALDI TOF/TOF MS), and found a total of 219 unique high confidence FF proteins through Swiss-Prot human database. The proteins he found were involved in complement, coagulation cascade, growth factor group, hormone group, immunity, and transportation [71]. Zakerkish *et al.* used mass spectrometry with the isobaric tags for relative and absolute quantification (iTRAQ) technology for isobaric tagging of peptides, which enables simultaneous identification and quantification of proteins, and analyzed the protein profiles of FF of the preovulatory and ovulatory phases and found 502 proteins, out of which 20 were overexpressed during ovulation. These proteins were inflammatory-related, coagulation factors, proteins in lipid metabolism, complement factors, and antioxidants. In addition, he found 5 proteins to be downregulated during ovulation, three of which were enzymes and two proteins of lipid metabolism and iron transport [72]. Poulsen *et al.* used liquid chromatography-mass spectrometry and found 400 proteins in FF, 40 of which showed significant change in their expression during ovulation. Among these were proteins involved in the immune and inflammatory system, secretion pathway, and proteins related to extracellular structure organization [73]. The involvement of the complement cascade in the folliculogenesis and oocyte maturation process was also shown by Jarkovska *et al*., who implemented 2DE, HPLC, and mass spectrometry in HFF of women undergoing IVF [74]. Angelucci *et al.* analyzed the follicular fluid and plasma from normo-ovulatory women undergoing assisted reproduction techniques, with 2DE and MALDI-TOF-MS, and found 183 HFF/plasma matched proteins and 27 unmatched. Many acute-phase proteins in high concentrations were identified, including transferrin, ceruloplasmin, afamin, hemopexin, haptoglobin, and plasma amyloid protein in the HFF, indicating that ovulation can be compared to an inflammatory event. Other proteins that were identified were some antioxidant enzymes such as catalase, superoxide dismutase, glutathione transferase, paraoxonase, heat shock protein 27, and protein disulfide isomerase. The above findings also indicate that during maturation the human follicle is protected against toxic injury due to oxidative stress [75]. Many studies have shown the importance of antioxidants in the normal oocyte growth. Nagy et al. showed that FF-HDL anti-oxidative function was related to a decrease in the odds of the oocyte undergoing normal fertilization [76]. Calonge *et al*. found that the activity of follicular fluid antioxidant enzymes was significantly lower in young women with reduced ovarian reserve compared with that in high responders and oocyte donors. Follicular fluid concentrations of oxidative stress marker malondialdehyde combined with 4-hydroxyalkenals and nitric oxide were higher in low responders than in high responders and oocyte donors [77]. In another study, Nishihara *et al*. showed that total GSH (glutathione) levels were lower in patients who had a low fertilization rate after ICSI, but it did not show a significant difference in pregnancy outcome. In addition, a total of 8-OHdG levels were higher in patients who had a low fertilization rate after ICSI and a low rate of good quality blastocysts. Total GSH and 8-OFdG in human FF may be potential markers for fertilization success in ART [78]. Lewandowska *et al.* used ultrafiltration to fractionate FF to high molecular weight (HMW) and low molecular weight (LMW) peptidome fractions. The HMW and LMW fractions were analyzed using LC-MS in sequential window acquisition of all theoretical (SWATH) data acquisition and processing methodology. A total of 158 proteins were identified out of which, 59 were never reported before as FF components. The concentrations of 11 proteins varied substantially among FF samples from single donors, and these proteins could be significant targets to identify biomarkers useful in oocyte quality assessment [79]**.** Bianchi *et al*. analysis of the follicular fluid suggests that effectors and inhibitors control and balance the induction and inhibition of inflammation, coagulation, and ECM degradation/remodeling. Such fine modulation of enzymatic activities plays an important role in follicle development and oocyte competence acquisition. Among the control proteins was alpha-1 antitrypsin, which is involved in 21 interconnections and may play a key role in balancing FF protease/anti-protease activity controlling ECM degradation, in inflammation, in wound response and coagulation cascade during follicle maturation, ovulation, and corpus luteum formation [80]. Klun *et al.* after implementing LC-MS/MS in oocytes found that tudor and KH domain-containing protein (TDRKH) is expressed in immature oocytes, while Wee2 (wee1-like protein kinase 2), PCNA (proliferating cell nuclear antigen), and DNMT1 (DNA (cytosine-5)-methyltransferase 1) were enriched in mature cells [81]. Bayasula *et al.* after implementing LC/MS/MS found that albumin and immunoglobulin families of proteins represent 80% of the total proteins. From the rest proteins that were identified, two were classified in the developmental process group, four in the signal transduction group, nine in the localization group, and 52 in the metabolic process group. Heparin sulfate proteoglycan percecan protein was upregulated in the group that resulted in the fertilized oocyte [82]. In the study of Zamah *et al.***,** 742 follicular fluid proteins were identified after implementation of high and low pH HPLC peptide separation, followed by mass spectrometry. Among them, 413 were not previously reported. The proteins belonged to insulin growth factor and insulin growth factor binding protein families, growth factor and related proteins, receptor signaling, defense/immunity, antiapoptotic proteins, matrix metalloproteinase related proteins, and complement activity. Moreover, after quantitative analysis, 17 follicular proteins were found at significantly altered levels between pre-hCG and post-hCG samples. These proteins belong to functional processes such as protease inhibition, inflammation, and cell adhesion [83].

### Potential biomarkers of female infertility

#### Potential biomarkers in follicular fluid

Hashmitabar *et al.* compared human follicular fluid from younger and older women with normal FSH levels with 2DE and MALDI-TOF-TOF mass spectrometry and identified twenty-three proteins differentially expressed. Five were downregulated in the older group which were serotransferrin, hemopexin precursor, complement C3, C4, and kininogen and are proteins involved in complement cascade pathway, immunity response, iron transport, and angiogenesis [84]. Estes *et al.* compared the proteome profile of follicular fluid in women under 32 years old between samples that did not lead to pregnancy and others that resulted in a live birth. After LC-MS/MS was performed, 11 potential protein candidates that were haptoglobin alpha, predominantly fetal expressed T1 domain, mitochondrial integrity genome (ATPase), apolipoprotein H (beta-2 glycoprotein I), dihydrolipoyl dehydrogenase, lysozyme C, fibrinogen alpha-chain, and immunoglobulin heavy chain V-III (region BRO) were increased in the live birth group, whereas antithrombin, vitamin D-binding protein, and complement 3 were decreased [85]**.** Kushnir *et al*., after depletion of abundant proteins from HFF samples and analysis with nano LC-QTOF, identified a total of 75 proteins, out of which 4 (paraoxonase 1, PRP6 pre-mRNA processing factor 6 homolog, complement component 6, and rho guanine nucleotide exchange factor 37) were present only in the IVF cycles that resulted in delivery, 7 proteins (complement component 2, hypothetical protein LOC64762, complement component c8 alpha-chain, apolipoprotein B, carboxypeptidase N, similar to Lg gamma-1 chain C region, alpha 2 globin) were present only in cycles that resulted in miscarriage, and finally, 2 proteins (growth inhibition and differentiation-related protein 88 and PHD finger protein 16) were identified only in IVF cycles that did not achieve pregnancy. In general, the proteins that were identified in the FF belong to the acute response signaling, coagulation system, neutroprotective role of THOP1, FXR/RXR activation, role of tissue factor, and growth hormone pathways. Proteins associated with biosynthesis were more abundant in the FF samples of oocytes that resulted in pregnancy, as well as 7 that were associated with steroidogenesis [86]. Severino *et al*. found 89 proteins, 30 of which were differentially expressed in hFF with successful compared to unsuccessful IVF outcomes. In particular, 2 were found to be downregulated. These were actin cytoplasmic 1 (ACTB), a structural constituent of the cytoskeleton, and tubulin polyglytamylase (TTLL7), involved in cell differentiation, while 28 were upregulated in hFFs with positive IVF outcomes [87]. Chen *et al****.***, after performing LC-MS/MS, identified 7 peptides as potential biomarkers for positive IVF outcomes. These were derived from insulin-like growth factor binding protein-5 (IGFBP5), alpha 2-antiplasmin (A2AP), complement component 3 (C3), inter-alpha-trypsin inhibitor heavy chain H1 (ITIH1), serum albumin (ALBU), protein diaphanous homolog 1, and plastin-3, which belong to different functional groups like growth factors, negative regulation of plasminogen activation, complement cascade, and stabilization of cumulus mass [88].

#### Biomarkers in granulosa/cumulus cells

Other studies have analyzed the granulosa/cumulus cells environment and have searched for potential biomarkers related to female infertility. Braga *et al.* compared protein expression of human cumulus cells of embryos that reached and did not reach the blastocyst stage. They found 87 different proteins in samples from the blastocyst and non-blastocyst groups, of which 30 were exclusively expressed in the blastocyst group and 19 in the non-blastocyst group. The proteins were binding proteins, enzymes, as well as structural proteins, transport proteins, contraction, and DNA repair proteins. Among the 72 proteins that were detected in the pregnancy group, 19 were exclusively expressed in the positive and 16 were exclusively expressed in the negative-pregnancy group [89]. Luddi *et al*., after implementing western blotting and immunofluorescence, showed the significant role of metalloproteinase, especially MMP2 (abbreviation) and MMP9, in fertilization. They found that MMP9 is expressed only in granulose cells, whereas MMP2 is more expressed in cumulus and granulose cells in cases of reduced ovarian response and decreased fertilization rate [90].

The above studies show that several molecular pathways in normal oocyte function are impaired in IVF cycles that did not have a positive outcome. Among the pathways are the inflammation pathway, complement and coagulation cascade, cell differentiation, and cytoskeleton organization (Table 2). Identifying biomarkers that indicate female infertility could help in the early prevention of an unsuccessful result or even in the monitoring of each cycle by a prospective therapeutic invasive approach, with the aim to achieve the desired outcome.

**Table 2:** Potential biomarkers of female infertility

Condition	Protein	Function	Up-/downregulated, unique in group	Reference
Older age group	Serotransferrin, hemopexin precursor, complement C3, C4, kininogen	Complement cascade, immunity response, iron transport, angiogenesis	Down	[84]
Positive IVF	Haptoglobin alpha, PFET1, MG11 (ATPase), B2GPI, DHD, lysozyme C, FGA IGHV3	Hemoglobin binding, enzymes	Up	[85]
Failed IVF	AT, VDBP, C3	Complement system	Down	[85]
Miscarriage	C2, C8A, APO-B, carboxypeptidase N, similar to Lg gamma-1 chain C region, alpha 2 globin	Complement system, FXR/RXR activation, and GH signaling	Unique	[86]
No pregnancy	GIDRP88, PHF16	Enzyme, metal ion binding	Unique	[86]
Successful IVF	ACTB, TTLL7	Structural constituent of cytoskeleton, cell differentiation	Down	[87]
Successful IVF	IGFBP5, A2AP, CO3, ITIH1, ALBU, protein diaphanous homolog 1 and plastin-3	Reduces activity of growth factors, negative regulation of plasminogen activation, complement cascade, stabilization of cumulus mass	Down	[88]

### Potential biomarkers for embryo quality

Until now, only a few studies have been conducted which search for biomarkers that indicate good embryo quality, either in an invasive manner where blastocoel fluid (blastocele fluid) is analyzed or in a non-invasive way (embryo secretome) through analysis of the embryo’s culture media.

Starting with the invasive studies, Katz-Jaffe *et al*., after lysis of blastocyst-stage embryos, studied the protein profile of the embryo using anion exchange chromatography, followed by SELDI-TOF-MS. Six proteins were found to be upregulated in arrested embryos compared to non-arrested ones. Candidate IDs for these proteins were heparin-binding EGF-like growth factor precursor (HB-EGF), cystatin-9-like precursor, CART/NADH-ubiquinone oxireductase, beta-catenin-interacting protein1, cytochrome c oxidase subunit, caspase-1 precursor, and inhibitor of growth protein 1 ING1-like tumor suppressor protein, X-linked. Many of these proteins play a role in implantation [91]. Poli *et al.* used an invasive shotgun proteomic analysis of blastocoel fluid to compare normal and aneuploid embryos and found two proteins to be differentially expressed among the two groups. These were GAPDH (glyceraldehyde 3-phosphate dehydrogenase) that was underexpressed in euploid and H2A (histone H2A) that was overexpressed in aneuploid embryos [92].

Moving on to non-invasive studies, Dyrlund *et al.* searched for biomarkers which are detectable in good quality embryo’s secretome. He used eight different commercial culture media and performed in-solution sample digestion with trypsin and LC-MS/MS after albumin depletion. A total of 110 proteins other than HSA (human serum albumin) were identified. Among them, eight have previously been suggested as biomarkers for embryonic viability. These are afamin, apolipoprotein A-I, epidermal growth factor receptor, haptoglobin, haptoglobin-related protein, peroxiredoxin-I, serotransferrin, and serum albumin. Based on biological processes, these proteins were grouped into three major groups: inflammatory response, innate immune response, and response to peptide hormone stimulus [93]. Kaihola *et al*. analyzed the proteome profile of embryo culture media using multiplex proximity extension assay (PEAs). He found that day-2 cultured embryos resulting in pregnancy after IVF treatment secreted significantly lower levels of caspase-3 in correlation with those that did not result in pregnancy. However, no differences were found in HRG (histidine-rich glycoprotein) levels between the two groups, although these were higher in culture media of embryos that reached the morula stage faster. The same analysis was also carried out for blastocysts, but no significant differences were found. The above results would make sense as caspase-3 is a protein involved in apoptosis (programmed cell death through DNA fragmentation). HRG inhibits the apoptotic effect of caspase-3 via interactions with thrombospondins. Normally, thrombospondins bind to CD36 and thereby initiate a cascade of events, ending in the activation of caspases to initiate apoptosis [94]. The lower levels of caspase-3 in high- versus low-quality blastocysts were also shown in the study of Lindgren *et al.* performing a multiplex proximity assay in human day-2 cryopreserved embryos. The study also found that embryos developing into blastocysts had higher levels of extracellular matrix metalloproteinase inducer protein (EMMPRIN) secreted in their culture media. Finally, the levels of VEGF-A, IL-6, and EMMPRIN were higher in embryos which reached the morula stage in a shorter time [95]. In another study by Katz-Jaffe *et al.*, protein expression of human embryos in culture media was again analyzed using anion exchange chromatography, followed by SELDI-TOF-MS. Ubiquitin was identified as a potential biomarker for embryo developmental potential [96]. Butler *et al*. performed ELISA and MALDI TOF-MS in embryos culture media and found that hCG, hCGh, and hCGΒ could be potential biomarkers of embryo viability and of their implantation potential [97]. Montsko *et al.* used LC coupled MS in culture media of in vitro fertilized embryos to correlate the alpha-1 chain of human haptoglobin (HPT) concentration and morphological score. He found that HPT concentration could predict the outcome of the embryo transfer [98]. In the study of Montsko *et al*., haptoglobin alpha-1 fragment was also found to be a potential biomarker for viable embryos. A significant correlation was also found among the presence of the peptide in the culture media and their achievement of pregnancy [99]. Mains *et al****.*** used embryo culture media to search for potential biomarkers showing good quality in embryos. He applied two-dimensional gel electrophoresis and mass spectrometry and found that apolipoprotein A1 is increased in culture media of blastocysts with higher morphologic grade [100]. McReynolds implemented LC-MS/MS to compare euploid and aneuploid blastocysts secretome and found nine potential biomarkers for aneuploidy, with the most significant being lipocalin-1 [101]. In the study by Domiquez *et al.*, culture media from blastocysts that were implanted and culture media from blastocysts that were not implanted were analyzed using protein array technology, and it was found that CXCL13 (BLC) and granulocyte-macrophage colony-stimulating factor (GM-CSF) was significantly decreased in the implanted blastocyst media compared to the non-implanted. The above proteins are involved in functions like a response to wounding, response to external stimulus/response to stress, response to pathogen or other organisms, inflammatory response, cell communication, immune response, and chemotaxis. Moreover, the soluble TNF receptor 1 and IL-10 were significantly increased, while MSP-A, also called hepatocyte growth factor-like (HGFL), SCF (stem cell factor), CXCL13 (C-X-C motif chemokine), TRAILR3 (tumor necrosis factor receptor superfamily member 10C), and MIP1b (macrophage inflammatory protein beta) was significantly decreased in culture media containing blastocysts (both implanted and non-implanted) in comparison with the control ones [102]. Cortezzi *et al.* implemented the nano-UPLC with nano-electrospray ionization (nano-ESI) in culture media samples from embryos that achieved and those that did not achieve pregnancy and found 18 proteins in the group that achieved implantation. Among them, protein Jumonji (JARID2), which composes a histone methyltransferase complex called polycomb chromatin methylation that silences many embryonic patterning genes, that normally serve as negative regulators of cell proliferation and may also be related to cell differentiation. Eleven more proteins were identified in the negative implantation group, with TSGA10 (testis-specific protein 10) being the most abundant protein. TSGA10 is a perinuclear protein which has structural activity and is detected in actively dividing and fetal differentiating tissues during developmental of mouse embryos [103]. Brison *et al*. tried to correlate the presence of specific amino acids with the potential of successful pregnancy after performing liquid chromatography. Three amino acids were identified, ASn, Gly and Leu, and were found to be significantly related to the achievement of pregnancy [104].

Pathways of the immune system, energy metabolism, apoptosis, structural pathways, and also implantation process play an important role in the regulation of embryo development. The list of the potential biomarkers of good embryo quality is presented below (Table 3). Proteomics could help in the single embryo transfer (sET) approach, as it could contribute to the selection of the best embryos, in combination with the morphological approach as well as the implementation of PGD.

**Table 3:** Proteomic biomarkers of competent embryos

Study group	Protein	Role	Up-/downregulated, unique in group	References
Arrested embryos	HB-EGF cystatin-9-like precursor, CART/NADH-ubiquinone oxireductase, beta-catenin-interacting protein1, cytochrome c oxidase subunit, CASP1pre, ING1, X-linked	Implantation	Up	[91]
Euploid embryos	GAPDH	Glycolysis pathway	Down	[92]
Aneuploid embryos	H2A	Structure of chromatin	Up	[92]
Embryonic viability	Afamin, APO A-I, EGFR, haptoglobin, HPR, peroxiredoxin-I, serotransferrin, ALBU	Innate immune response, inflammatory response	Up	[93]
Day 2 embryos, blastocysts resulting in pregnancy	Caspase-3	Apoptosis	Down	[94]
Faster in the morula stage	VEGF-A, IL-6, EMMPRIN	Implantation	Up	[95]
Embryo developmental potential	Ubiquitin	Implantation	Up	[96]
Embryo viability	hCG, hCGh, and hCGΒ	Implantation	Up	[97]
Viable embryos	Haptoglobin alpha-1 fragment	Acute-phase reactant	Down	[98]
Good morphological grade	Apolipoprotein A1	Cholesterol transport	Up	[100]
Aneuploid embryos	Lipocalin-1	Overproduced In stress, infection, inflammation	Up	[101]
Implanted blastocyst group	CXCL13 (BLC), GMCSF	Immune response	Down	[102]
Implanted embryo group	JARID2	Negative regulator of cell proliferation.	Unique	[103]
Non-implanted embryo group	TSGA10	Perinuclear protein which has structural activity	Unique	[103]
Culture media of successful IVF	Gly, Leu	Aminoacids	Down	[104]
	Asn	Aminoacids	Up	[104]

### Endometrial proteomics

The identification of a receptive endometrium could contribute to the prevention of implantation failure and pregnancy loss and could therefore lead to an increase in ART success rates. There is a need for a practical, non-invasive test to predict the receptivity of the endometrium for embryo implantation. The crosstalk between the embryo and the endometrium is time- and location-sensitive, occurring during a short time span, usually between days 16 and 22 of a 28-day normal menstrual cycle, known as the “window of implantation” 5–10 days after the luteinizing hormone (LH) surge [105]. Currently, there are no markers of endometrial receptivity [106]. The proteomics approach to endometrial receptivity can either be studied by analyzing endometrial tissue or uterine fluid.

#### Endometrial tissue

Studies that utilized endometrial tissue to compare receptive and non-receptive endometrium are presented below. Hood *et al.* performed a proteomic analysis of the endometrial tissue using LC-MS/MS, followed by immunochemistry, in order to compare the proliferative and secretory endometrium. A total of 318 proteins were found to be differentially expressed between the two phases in the epithelial cells and 19 in the stroma compartment. Proteins identified from glandular epithelial cells included progesterone receptor B, expressed in the proliferative phase, and glycodelin A (PAEP), expressed in the secretory phase. In addition, CPM, paladin (PALLD), minichromosome maintenance complex component 6 (MCM6), ENPP3, periplakin (PPL), homogentisate 1,2-dioxygenase (HGD), and polymeric immunoglobulin receptor (PIGR) were also significantly differentially abundant in the glandular epithelium of the proliferative as compared to the secretory phase. The above proteins indicate an upregulation of cellular growth and proliferation molecular pathways in the proliferative compared to the secretory endometrium [107]. DeSouza *et al*. used isotope-coded affinity tags, three stages of chromatographic separation, and online tandem mass spectrometry (MS/MS) to also analyze the proteomic profile differences between the secretory and proliferate endometrial tissue. He found five proteins to be consistently differentially expressed between the two phases, with glutamate NMDA receptor subunit zeta 1 precursor and FRAT1 being the most frequent in the secretory endometrium. The first protein is known to be involved with synaptic plasticity in neurons. In a recent paper, it is suggested that it may also play a role in glutamate-mediated toxicity to mitochondria, leading to apoptosis. FRATI is known to inhibit c-Jun activity, thereby inhibiting subsequent apoptosis [108]. Differences in mid-secretory and proliferative phases of the endometrial tissue were also found in the study of Parmar *et al.*, who used two-dimensional protein maps, followed by MALDI-TOF-TOF to compare MSE (mid-secretory endometrium) with PROE (proliferative phase endometrial tissues) as well as with MSU (mid-secretory phase uterine fluids) and found Calreticulin precursor, fibrinogen, adenylate kinase isoenzyme 5 (KAD5), and transferrin to be upregulated in the proliferative phase endometrium. The above proteins participate in cellular activities such as calcium-binding, blood clotting, energy metabolism, and blood plasma protein, respectively, while annexin V (ANXA5), peroxidoxin 6 (PRDX6), a1-antitrypsin (AAT), and creatine kinase were upregulated in the mid-secretory phase, whose functions include apoptosis, antioxidant, protease inhibitor, and energy metabolism [109]. Another study by Chen et al. that aimed to identify proteins that were differentially expressed in the human endometrium tissue between proliferative and secretory phase with 2D differential in-gel electrophoresis (DIGE) showed enhanced expression of proteins in the secretory endometrium. The differentially expressed isoforms of the same proteins were identified by MALDI-TOF/TOF MS. These isoforms belonged to 4 proteins, three of which were increased in the mid-secretory phase. These were annexin A4 (ANXA4), keratin 8 (KRT8), and heat shock protein beta 1 (HSPB1), while albumin (ALBU) was decreased. Their functions are differentiation, apoptosis and inhibition of proliferation (ANXA4), cellular assembly and organization (KRT8), and heat stress (HSPB1) [110]. Annexin A2 (ANXA2) and stathmin I (STMNI) were found to be differentially expressed in non-receptive (day 2) versus receptive (day 7) endometrium in the study of Dominguez *et al.* after implementation of 2DE and MALDI-MS on endometrial tissue. Both proteins play a role in cytoskeletal development. Stathmin I is also involved in the intracellular signaling cascade [111]. Garrido-Gomez *et al*., after performing DIGE and MALDI mass spectrometry in receptive and non-receptive endometrial biopsies, found 24 differentially expressed proteins. He applied in silico analysis and identified the pathways that were most different between the two groups. These were the carbohydrate biosynthetic pathway and the rearrangement of the cytoskeleton pathway. After immunochemistry was performed, it was found that PGRMCI (progesterone receptor membrane component I) and ANXA6 (Annexin A6) play an important role in endometrial receptivity [112]. Another study by Berkova *et al.*, who performed HPLC and immunoblotting, found that the concentration of haptoglobin was significantly higher in deciduas graviditatis in comparison with non-pregnant endometrial tissue and higher in the stroma in contrast to the epithelium of the proliferative endometrium. In the secretory phase, it was found to be in moderate concentrations in the stroma in contrast with the epithelium. Haptoglobin in the uterus may bind with hemoglobin but could also be involved in the multi-factorial mechanism protecting the fetus from a maternal allograft-like immune response [113].

#### Endometrial fluid

More non-invasive studies analyzing the proteome of the uterine fluid have been conducted. Casado-Vela *et al*. set three different proteomic approaches (in-solution tryptic digestion and SDS-PAGE, followed by HPLC-MS/MS and 2D-PAGE, followed by MALDI-TOF/TOF) in order to analyze the proteomic profile of endometrial fluid and found in total 803 proteins, including albumin, IgGs, transferrin, fibrinogen, antitrypsin, complement C3, haptoglobin, apolipoprotein, ceruloplasmin, and complement factor B [114]. Matorras *et al.*, in another study, analyzed endometrial fluid right before embryo transfer after 2DE MS/MS and found 23 proteins differentially expressed between successful embryo implantation cycles and failed cycles. Most of these proteins were downregulated in the group that achieved implantation, and these were heat shock cognate (HSP cognate), heat shock (HSP), plastin-2, protein disulfide-isomerase A3 (PDIA3), arginase-1, F-actin-capping protein subunit alpha-1 (CAPZA-1), putative beta actin-like protein 3 (ACTBL3), actin, cytoplasmic1, proteasome subunit beta type 4 (PSMB4), protein deglycase DJ-1, (Parkinson disease protein7), superoxide dismutase[Mn], mitochondrial (SOD2), cell division control protein 42 homolog (CDC42), cofilin-1, stathmin, myeloid-derived growth factor (MYDGF), tubulin-specific chaperone A (TBCA), glyceraldehydes-3-phosphate dehydrogenase (GAP-DH), F-actin capping protein subunit beta (CAPZB), and annexin A2 (ANXA2). The above proteins are involved in cell growth, signal transduction, metabolism, cell communication, blood coagulation, barbed-end actin filament capping, and regulation of nucleobase, nucleoside, nucleotide, and nucleic acid metabolism. Four proteins were upregulated, which were catalase, serum albumin (ALBU), serotransferrin, and Lg kappa chain V. These proteins are involved in immune response and transport. The last three should be considered to have a blood origin or nonspecific source [115]. Hannan *et al*. analyzed the endometrial secretions and compared receptive and non-receptive states in fertile and infertile women after 2D-DiGE and found 7 spots significantly decreased in the MP (mid proliferative) phase compared to the MS (mid-secretory) of the endometrium and 18 spots between fertile and infertile women. Moreover, after immunostaining of the endometrial tissue, antithrombin III was found to be localized in glandular and luminal epithelium with higher levels in MP in contrast to the MS phase. The higher concentrations of antithrombin iii were also found in the infertile endometrium in comparison to the fertile one. Another protein that was found to be upregulated in the MS phase and in moderate levels in the MP phase was a2 macroglobulin; however, there was no difference in the intensity of A2macroglobulin immunostaining between fertile and infertile women. ANT3 has anti-inflammatory properties, while A2M deactivates of matrix metalloproteinases (MMPs), limiting trophoblast invasion [106]. Kasvandik *et al*. analyzed the proteome of early (ESE) and mid-secretory endometrium (MSE) of fertile and infertile women after performing LC/MS/MS and found 367 proteins that undergo significant proteomic changes while transitioning from the early to mid-secretory endometrial phase. Twenty-one proteins were found to display similar levels between control ESE and RIF (repeated implantation failure) MSE, indicating the displacement of WOI. Four proteins had similar levels to control ESE than to control MSE, potentially indicating a pre-receptive EM in the RIF cohort [116]. Scotchie et al. analyzed the luteal endometrial secretome (lh+4, lh+9) by performing 2DE and MS/MS and found 82 proteins to be differentially expressed. Increased expression in the secretory phase showed proteins involved in host defense and molecule transport. Among them were haptoglobin precursor, anti-TNFα antibody, apolipoprotein A1 fragment, transferrin precursor, and vitamin D binding protein variant, while proteins with decreased expression had many functions, like apoptosis regulation, cell proliferation, and host defense. Among them were heat shock protein b1, clusterin precursor, cofilin1, haptoglobin precursor, and others [117]. Al-Rumaih *et al.* attempted to identify uterine markers in early and late proliferative phases of the menstrual cycle with respect to estrogen levels using 2D-PAGE protein maps and found that nineteen spots were differentially expressed in the two groups; five of them were identified and were also increased in the high E2 group. These were serotransferrin (STF), hemopexin precursor, fibrinogen β chain, a-1 antichymotrypsin (AACT), and complement component C4. The first two are involved in the transport of iron. Hemopexin is also involved in acute-phase reactions. Fibrinogen and AACT are involved in blood coagulation, complement activation, programmed cell death, and development [118]. Fitzgerald *et al*. analyzed with LC-MS/MS endometrial fluid of the proliferative phase from fertile and infertile women and found four proteins to be downregulated in the infertile women. These were extracellular matrix protein 1 (ECM1), transforming growth factor–beta-induced protein ig-h3 (TGFB1), secreted frizzled-related protein 4 (SFRP4), and CD44 antigen. Their role is negative regulation of signaling, cell communication, and tissue development. Upregulated were found to be two proteins, protein-glutamine gamma-glutamyltransferase 2 (TGM2) and Lg gamma-4 chain C region (IGHG4). In addition, seven proteins were unique in the fertile group. These were filamin A (FLNA), pregnancy zone protein (PZP), oviduct specific glycoprotein (OVGP1), endoplasmin (HSP90B1), annexin A6 (ANXA6), 40s ribosomal protein S3 (RPS3), and septin-2 (SEPT2). These proteins take part in cilium assembly, actin filament organization, and macromolecule catabolic process. Finally, six proteins were found to be unique in the infertile group; these were neuroblast differentiation-associated protein (AHNAK), isoaspartyl peptidase/L-asparaginase (ASRGL1), UMP-CMP kinase (CMPK1), phosphoglucomutase-1 (PGM1), cornulin (CRNN), and suprabasin (SBSN). These proteins are involved in carbohydrate metabolism and other cellular processes [119]. Bhutada *et al*. used endometrial fluid and tissue of the early secretory or secretory phase. After iTRAQ analysis and immunoblotting, he identified HMGBI (amphoterin), a DNA binding non-histone protein, in pre-receptive and receptive endometrium. The protein was downregulated in the receptive phase compared to the pre-receptive phase of the endometrium [120]. Gillot et al., after analysis of endometrial flushing of the proliferative phase endometrium from thirty-one women undergoing assisted reproduction, using 2DPAGE, found a statistical difference in the expression of two leucine-rich alpha2-glycoprotein (LRG) isoforms, which were higher in women who got pregnant. These isoforms may be involved in the infiltration of decidua by uterine natural killer cells, which actually differentiate into granular forms during early pregnancy [121]. Azkargorta *et al*. analyzed with LC-MS endometrial fluid between cycles that achieved and some that did not achieve implantation and found 212 differentially expressed proteins. After characterization of the proteins in non-pregnant women, these were found to be involved in pathways such as innate immune response, inflammation, obesity, infection, human antimicrobial proteins, cell-cell adhesion, immune response, response to stress, and oxidation-reduction processes. A subset of stress and immune response-related proteins are part of the complement signaling cascade. This result may imply intrinsic deregulation of the complement signaling in women in whom no implantation occurred, but could also reflect the activation of antimicrobial response in these patients. After analysis with the IPA program, it was found that in women where implantation was not achieved, an increase in organismal death and inflammation-related processes as well as inhibition of cell movement and migration was detectable. The biomarker discovery approach revealed that glycogen phosphorylase, brain form (PYGB) was downregulated in endometrial fluid aspirate, where no implantation occurred and it could be an interesting candidate for the discrimination between positive and negative cycles. Glycogen phosphorylases are involved in glycogenolysis, catalyzing the rate-limiting step in the process, but they are also linked with the oxidative stress response. Actually, it is already shown that reduced glycogen phosphorylase activity is related to increased oxidative stress [122].

The protein pathways that are mainly affected and, as a result, show an important role in the achievement of implantation are those of cellular growth, apoptosis, skeletal development, differentiation, energy metabolism, immune response, and oxidative stress (Table 4). The above pathways are in agreement with the cellular pathways behind implantation failure or pregnancy loss, which among others, involve the immune system and the coagulation system [123, 124]. The measurement of endometrial biomarkers could be carried out after the collection of some uterine discharge with a cotton applicator early during the process of IVF.

**Table 4:** Protein biomarkers of endometrial receptivity

Study group	Protein	Role	Up-/downregulated, unique in group	References
Proliferative endometrium	Progesterone receptor B, CPM, PALLD, MCM6, ENPP3, PPL, HGD, and PIGR	Cellular growth, proliferation	Up	[107]
Secretory endometrium	PAEP	Inhibition of immune response	Up	[107]
Secretory endometrium	NMDA receptor subunit zeta 1 precursor, FRAT1	Leading to apoptosis, inhibiting subsequent apoptosis	Up	[108]
Proliferative endometrium	Calreticulin precursor, fibrinogen, KAD5, transferrin	Calcium-binding protein, blood-clotting protein, energy metabolism, and blood plasma protein	Up	[109]
Secretory endometrium	ANXA5, PRDX6, AAT, creatine kinase	Apoptosis, antioxidant, protease inhibitor, and energy metabolism.	Up	[109]
Secretory endometrium	ANXA4, KRT8, and HSPB1	Differentiation, apoptosis, inhibition of proliferation, cellular assembly and organization, heat stress	Up	[110]
Secretory endometrium	ALBU	Binding and transportation	Down	[110]
Receptive endometrium	ANXA2	Skeletal development	Up	[111]
Receptive endometrium	STMNI	Cytoskeleton, intracellular signaling cascade	Down	[111]
Receptive endometrium	PGRMCI, ANXA6	Metabolism, rearrangement of cytoskeleton	Down	[112]
Proliferative endometrium	Haptoglobin	Binding of hemoglobin, multi-factorial mechanism protecting the fetus from a maternal allograft-like immune response	Up	[113]
Successful implantation	HSP cognate, HSP, plastin-2, PDIA3, arginase-1, CAPZA-1, ACTBL3, actin, cytoplasmic1, PSMB4, protein deglycase DJ-1, SOD2, CDC42, stathmin, MYDGF, TBCA, GAP-DH, CAPZB, ANXA2	Cell growth, signal transduction, metabolism, cell communication, blood coagulation, barbed-end actin filament capping and regulation of nucleobase, nucleoside, nucleotide and nucleic acid metabolism	Down	[115]
	Catalase, ALBU, serotransferrin, Lg kappa chain V	Transport, metabolism	Up	[115]
Proliferative endometrium	ANT3, A2M	Anti-inflammatory properties, deactivation of MMPs and limits trophoblast invasion	Up	[106]
Secretory endometrium	Haptoglobin precursoranti-TNF a antibody, apolipoprotein A1 fragment, transferrin precursor, vitamin D binding protein variant	Host defense and molecule transport	Up	[117]
	Heat shock protein b1, clusterin precursor, cofilin1, haptoglobin precursor, and other	Apoptosis regulation, cell proliferation, host defense	Down	[117]
Increased in high E2 group	STF, hemopexin, fibrinogen β chain, AACT, C4	Iron transport, acute-phase reaction, blood coagulation, complement activation, programmed cell death, development	Up	[118]
Infertile group	ECM1, TGFB1, SFRP4, CD44	Negative regulation of signaling, cell communication, tissue development	Down	[119]
Infertile group	TGM2, IGHG4	Angiogenesis, wound healing, cell differentiation, immune response	Up	[119]
Fertile group	FLNA, PZP, OVGP1, HSP90B1, ANXA6, RPS3, SEPT2	Cilium assembly, actin filament organization, macromolecule catabolic process	Unique	[119]
Infertile group	AHNAK, ASRGL1, CMPK1, PGM1, CRNN, SBSN	Carbohydrate metabolism, cellular process	Unique	[119]
Pregnant group	LRG isoforms	Infiltration of decidua by uterine natural killer cells	Up	[121]
Receptive endometrium	HMGBI	DNA binding non-histone protein	Down	[120]
No implantation group	PYGB	Glycogenolysis, oxidative stress response	Down	[122]

### Commentary and outlook

Proteomics is a recently developed field which seeks for protein biomarkers that are the content of a cell, tissue, or organism [21]. Such biomarkers could help predict, diagnose, and monitor in a personalized way human pathologies such as infertility and could contribute to the achievement of the desirable outcome [23]. Despite its extra cost, proteomics could benefit each individual by minimizing the number of IVF cycles that they have to undergo in order to achieve the desired outcome and, at the same time, in avoiding the phycological discomfort and disappointment that each unsuccessful cycle leads to. Moreover, since the incorporation of mass spectrometry in proteomic technologies, the cost of this technology has significantly decreased.

Furthermore, it is important to mention that omics represent non-invasive approaches in comparison to other invasive assessments such as PGD, or the morphological evaluation under the microscope (which is still not replaced in most of IVF laboratories from time-lapse technology), which may prove to be a significant advantage of these technologies.

Apart from proteomics, it is important to also refer to other omic approaches, the application of which, as well as their combination with proteomics, could significantly add to the comprehension of biological processes and molecular functions in the reproduction field and benefit the IVF community. More precisely, the field of omics includes genomics which studies the DNA sequences in an organism, tissue, or cell, transcriptomics which studies their transcription products, epigenomics which assesses epigenomic modifications, and metabolomics which focuses on the metabolites that are produced after normal cell processes. Other new omics approaches include exomics (analysis of exons), secretomics (analysis of secreted products), and lipidomics (large-scale analysis of whole lipid species) [125]. The combination of the above approaches, named as multi-omics, aims to identify molecular markers that are associated with biological processes by revealing the regulatory units across diverse omics layers (obtained from DNA, RNA, proteins, and metabolites) and assists in the understanding of biological processes and molecular functions. Multi-omics can contribute to the identification of predictive and prognostic biomarkers and even novel drug targets in the era of precision medicine [126].

## Conclusion

Despite the great numbers of live births that have been achieved through ART, there is still potential for increasing the success rates. Up to now, ART success rates remain suboptimal ART procedures have a 70% possibility of failure. The field of proteomics, which is a recently developed field, could provide the ART community with important insights on a molecular level and help discover the physiological processes behind human reproduction. This is achieved by studying the interactions that take place between the sperm, the oocyte, the embryo, and the uterus. Proteomics utilizes the application of technologies for the identification and quantification of overall proteins (biomarkers) that are the content of a cell, tissue, or organism. More precisely, proteomics identifies the alterations in the expression profile of key proteins which are involved in the cellular and molecular pathways behind normal sperm, oocyte, embryo, and endometrial tissue/fluid interactions and function. Such biomarkers could help predict the outcome, prevent failure, and monitor in a personalized manner in vitro fertilization (IVF) cycles, and it could in such a way contribute to the achievement of the desirable IVF outcome and improve ART success rates worldwide, having a major impact on the successful clinical management of infertile patients.

### Recommendations

It is clear that the PPPM approach in the IVF field could be proven to be very beneficial, as it could assist in the increase of the IVF success rates. IVF is a field that requires an individualized approach to the patient, which is the main focus of PPPM medicine. Moreover, the ability to predict the IVF cycle outcome and prevent an undesirable result could offer a lot to the IVF community [127]. Proteomics has enabled the research community to identify differentially expressed proteomes in fertile and infertile individuals. This contributes to our understanding of molecular activities which influence the normal and abnormal biological activity of sperm, oocyte, embryo, and the endometrium. The studies gathered in this review show differentially expressed proteins among fertile and infertile individuals. Interestingly enough, in all four biological samples (sperm, oocyte, embryos, endometrium), proteins that have a key role in activities such as membrane transport, structure, energy, and metabolism are abounded in fertile groups, while proteins of the immune response are higher in quantity in infertile groups. More precisely, the analysis of sperm proteomics showed membrane transport proteins like ANXA proteins and proteins involved in capacitation like CLU protein to be more abundant in infertile groups, while PIP which is also involved in capacitation to be abundant in the fertile population. The immune response is also shown to play a significant role in infertility as proteins related to it are more abundant in infertile individuals. Moreover, the relationship between the immune response and the inability to successfully achieve and complete pregnancy is also clear in the studies of follicular fluid that no pregnancy was achieved. The results of proteomic studies on embryos that were successfully implanted in human endometrium showed increased apolipoprotein A1 levels, while immune response-related proteins were once again higher in the infertile groups. Finally, with regards to the endometrium, the studies show that receptive endometrium is more abundant in proteins related to skeletal development, while secretory endometrium has a higher quantity of proteins related to apoptosis. The above findings are still not clear as there are many studies that contradict them. Subsequently, it would be too early to recognize the above proteins as potential candidate biomarkers. It is understandable that more studies need to be conducted in order to clearly identify proteins as biomarkers of human infertility and be able to predict and IVF outcomes, prevent an unsuccessful cycle, and monitor them in a personalized manner in order to achieve the best possible outcome and have the desired result.

## Data Availability

The datasets generated during and/or analyzed during the current study are available from the corresponding author on reasonable request.
